# Role of community health service programs in navigating the medical ethical slippery slope—a 10-year retrospective study among medical students from southern China

**DOI:** 10.1186/s12909-019-1652-5

**Published:** 2019-07-01

**Authors:** Guanhua Fan, Zhenhua Lin, Yizhen Luo, Maohuai Chen, Liping Li

**Affiliations:** 1 Shantou University Medical College, 22 Xin Ling Road, Shantou, 515041 China; 2 The First Affiliated Hospital of Shantou University Medical College, 57 Chang Ping Road, Shantou, 515041 China; 3 Shenzhen Children’s Hospital, 7019 Yitian Road, Shenzhen, 518172 China

**Keywords:** Community medicine, Community health service, Community health education, Health promotion in rural areas, Medical student, Cognitive level, Medical ethics

## Abstract

**Background:**

For promoting autonomous learning motivation, the learning effect of community-oriented service is beneficial, because through community participation and service, students can transfer their implicit cognition of ethics into explicit cognition, leading to the cultivation of a sympathetic partnership between the community and medical students. Despite the proven benefits of medical students’ community health service (CHS) in Western countries, CHS programs designed for medical students are not well established in mainland China, and their effects on medical students’ ethical cognition are largely unknown. This study evaluated the effects of CHS programs on the ethical cognition of medical students.

**Methods:**

A cross-sectional study was conducted on third- and fourth-year medical students and graduates working at Shantou University Medical College by using a self-administered anonymous questionnaire. Through interviews, we applied a thematic approach to analyze the responses of the participating students. The questionnaire adopted in this study was revised based on a review of the literature on medical ethics in medical students and on the CHS environment in China. The reviewed questionnaires included an evaluation questionnaire on cultivating medical ethics in a CHS context, and questionnaires used to explore the cultivation and transformation of medical ethics in medical students during the preclinical period.

**Results:**

A total of 361 (54.4%) undergraduate medical students and 302 (45.6%) graduates participated in this survey. Significant differences were observed in self-evaluation of the cognitive development of ethics between those who had participated in CHS programs 1–5 times and those who had participated > 6 times. The successful identification of accepting money from the patients under the table as unethical behavior significantly differed (*p* = .031) among the graduates but not (*p* = .567) among the undergraduate students. The participants expressed the positive impact of CHS programs on their self-development.

**Conclusion:**

CHS programs can be widely applied in medical education in China. This educational strategy, which supports medical professionalism and incorporates humanitarian behavior as a complement to learning, should be encouraged and promoted nationally.

## Background

Chinese medical education and practice have long ignored the cultivation of professionalism in medical students. An article entitled “Lessons from the East—China’s rapidly evolving health care system,” [1] published in the *New England Journal of Medicine* in in April 2015, states that the cultivation of Chinese doctors’ professionalism, which concerns physicians, patients, hospitals, and medical colleges, has been an important part of the Chinese medicine revolution. However, as a solid foundation of the modern health system, the function of professionalism has not been paid sufficient attention. The lack of general professionalism has made the Chinese medical environment complicated and has made it difficult to cultivate leaders and trustworthy medical staff. The findings of this study indicate that the usefulness of a community health service (CHS) lies in whether medical students can apply what they have learned in the classroom to their practices within the CHS. Moreover, through reflection, students’ civic awareness can be improved [2]; thus, reflection has become an important component of medical education worldwide and is being applied in the teaching processes of many colleges and universities. The use of CHS programs in academic study can help students in more clearly understanding the needs of patients and in more effectively solving practical problems [3–5].

CHS activities improve students’ awareness regarding the importance of the long-term management of chronic diseases. In China, severe chronic diseases, including cancer, have become the leading cause of mortality [6]. Residence in a rural area, a low yearly family income, and a low educational level were reported as behavioral risk factors related to severe chronic diseases [7]. In China, more than 60% of the population lives in rural areas, and the health care and public health facilities are still relatively underdeveloped [8, 9]. Long-term mechanisms, including cooperation between the government and media, should therefore be established to promote countrywide CHS programs for the benefit of medical students and economically disadvantaged populations in mainland China [10].

Social cognitive theory (SCT) assumes that the learning behavior of humans mostly occurs in the social environment. In a dynamic society, personal factors, the environment, and human behaviors continually interact [11], indicating that personal behaviors are the result of reciprocal determinism between humans’ cognition and environment. In other words, the mutual effect of the environment, behaviors, and personal factors, such as cognition, is persistent [12], and humans learn social behaviors and conduct through observing other people [13]. SCT explains the reciprocal action of CHS on medical students’ behaviors, conduct, and environment. However, the identification of a method for assessing the efficiency of community medical service is challenging [14]. If we intend to particularly emphasize the participants’ learning efficiency, quantitative research methods can be used. To date, the most commonly used method for evaluating the efficiency of CHS is self-evaluation reports [15].

Community medical activities make useful contributions to medical education, particularly by exposing medical students to CHS and providing first-hand experience of common environmental and living health concerns [16–19]. This promotes the development of care for others and facilitates essential abilities, such as communication, leadership, teamwork, understanding of health, and development of cognitive–emotional dimensions [20–22].

Holistic medicine and medical students’ professionalism require the provision of respect, care, and support through empathizing with and understanding those in need, which is an essential ability for current medical students [23]. However, the deficient cultivation of professionalism is the greatest challenge in Chinese medical education [24]. In summary, teaching students to first be an ethical person and then an ethical professional should be a goal of Chinese medical education. In mainland China, CHS programs are still in the exploratory stages, especially in terms of their application in medical education. Shantou University Medical College (SUMC) has incorporated CHS into medical education in mainland China since 2004. China has an immense agricultural population with considerable social expenditure and income inequality, especially between the urban and rural areas [25]. Hence, CHS programs are provided for the low-income sectors of society, particularly in economically underdeveloped areas. These CHS programs are funded by the Li Ka Shing Foundation and supported collectively through regular clinics, home-based service units, and medical art performers [26]. The learning objectives of the service programs of SUMC include community health assessment, risk behavior change, assistance with personal health services (e.g., for patients with advanced cancer and their families), advocacy and policy change, environmental interventions, medical training for rural communities, partnership-building for children and older adults in the families of cancer patients, and program evaluation [27, 28]. The learning objectives are interwoven across the undergraduate and graduate students’ curricula as they gain skills in creating, delivering, and evaluating evidence-based public health approaches at the community level [29] (Table 1).

**Table 1:** Main platforms for CHS in SUMC

Platform	Content and outcome offers of CHS activities
National hospice plans of Li Ka Shing Foundation (founded in 1998)	Poor patients with advanced tumors are provided with physical, mental, social, and spiritual types of analgesic treatment and psychological support through home-based services. The quality of life status of patients with advanced cancer is considered the main outcome measure for palliative care services [30, 31]. CHS in hospices, as a special phase of palliative care, provides interprofessional and multidimensional care for patients with advanced cancer and their families [32–34].
National health-related antipoverty project of Li Ka Shing Foundation (founded in 1998)	The travel of medical students to poor, rural areas, including the provinces of Fujian, Jiangxi, Guangdong, Guangxi, Yunnan, and Tibet, is organized to execute health-related antipoverty activities. Medical ethics education and the training of a large number of local medical personnel are promoted.

To cultivate medical talent, SUMC offers a 8-year course, which consists of 5 years of preclinical courses and 3 years of clinical rotations. The main participants in CHS programs in this study were third- and fourth-year medical students. These programs were held on weekends to avoid conflict with the students’ regular school learning.

Although participation was voluntary and not awarded with class-hour credits, most of the students were willing to participate in their spare time. All participants were trained by clinical teachers for acquiring knowledge on chronic diseases and the local cultural and social characteristics. In the mornings, volunteer experts provided clinical service, and the students joined them during patient encounters, if necessary. The students provided patients with education and physical examinations, including free health screening for high blood pressure, obesity evaluation (body mass index measurement), random blood glucose tests, and eye examinations, at their own discretion. During the service activities, the students distributed self-designed leaflets describing common diseases to the local villagers. During home-based service in the afternoons, the students, accompanied by experts, interacted more closely with the patients’ families to obtain first-hand experience of their common environmental and living health concerns. Each semester, the students sent performers with distinct talents and resources to the countryside. In addition to traditional performances such as singing, dancing, instrumental music, comedy, and magic shows, they offered humorous performances regarding first aid techniques, including demonstrations for fire emergencies, electric shock treatment, and cardiopulmonary resuscitation. After the programs, feedback and comments were obtained through e-mail, telephone, and postal mail.

Services and learning complement each other. These activities offer medical students the opportunity to provide medical service to rural communities, many of which have limited access to basic health care. CHS is an educational laboratory for the application of knowledge [33, 34]. Through these programs, students’ social responsibility and awareness are improved, and poor, rural populations can acquire a high quality of medical services. Although CHS programs have been integrated into medical education in mainland China for 10 years, the effects of CHS programs on the cognition of medical ethics had not been evaluated. Therefore, the emphasis of this study was on cognition, or students’ thoughts regarding medical ethics before and after they participated in CHS programs, rather than on knowledge development [35]. The specific objective of this study was to determine the effects of CHS programs so that the quality of undergraduate medical education and the concept of CHS in China can be improved.

The perceptions of medical students toward medical ethics have been studied previously, with attributes such as altruism, autonomy, caring, and compassion often listed by students as the most important with regard to medical ethics. However, others are often emphasized, such as leadership, excellence, creativity, motivation, values, aspirations, self-confidence, and initiative [36, 37]. Medical ethics cognition is a concept with high cultural sensitivity that develops with time and changes according to social values. Consequently, the differences between Western and East Asian cultures can be stark. Based on the medical situation in mainland China and due to cultural differences, medical ethics cognition should be explored and developed while accounting for local cultural characteristics. Thus, in addition to CHSs, Chinese medical students’ understanding and rationale regarding medical ethics should be explored, which is a topic on which no systematic research has been conducted previously.

## Methods

### Data collection

This section has been divided into Data Collection and Data Processing and Results subsections to illustrate our research process.

The questionnaire adopted in this study was revised based on a review of the literature on medical ethics among medical students and the CHS environment in China. These included a questionnaire used to evaluate the cultivation of medical ethics in a CHS context [38], and questionnaires used to explore the cultivation and transformation of medical ethics in medical students during the preclinical period [39–44]. After the questionnaire for this study was compiled, experts were invited to examine its content validity, and the questionnaire was pretested based on the experts’ revisions. After the questionnaire was finalized, it was used to conduct on-site surveys of the respondents.

In addition to benefitting the community, CHS promotes the development of students’ cognitive ability, morality levels, self-awareness, and self-confidence [45]. On the basis of previous studies, this retrospective cross-sectional descriptive study utilized a mixture of qualitative and quantitative methods. We mainly focused on the effects of CHS programs on the cognition of medical ethics and therefore designed a self-administered anonymous questionnaire containing the following items:

- demographic characteristics,
- self-evaluation of ethical sensitivity development, and
- self-reports of ethical behavior.

All investigators were given uniform training. The questionnaires were hand-delivered to all participating medical students from the school and physicians who had graduated and worked at the hospital.

Comparisons of categorical data were performed using the Pearson chi-square test. The Statistical Package for Social Sciences (IBM SPSS Statistics 19.0) was used for data analysis, and the level of statistical significance was set at the conventional *p* < .05.

The questionnaire formed the basis of the content for the interview, which consisted of a series of open-ended questions regarding the impact of CHS on the cognitive development of the participants. The interview was conducted by two trained interviewers, and all participants provided verbal informed consent [46, 47]. We audio-recorded all interviews, and all recordings were transcribed into text by two authors. The interviews were transcribed verbatim and thematically analyzed using NVIVO 10 software.

### Data processing and results

**Fig. 1:**
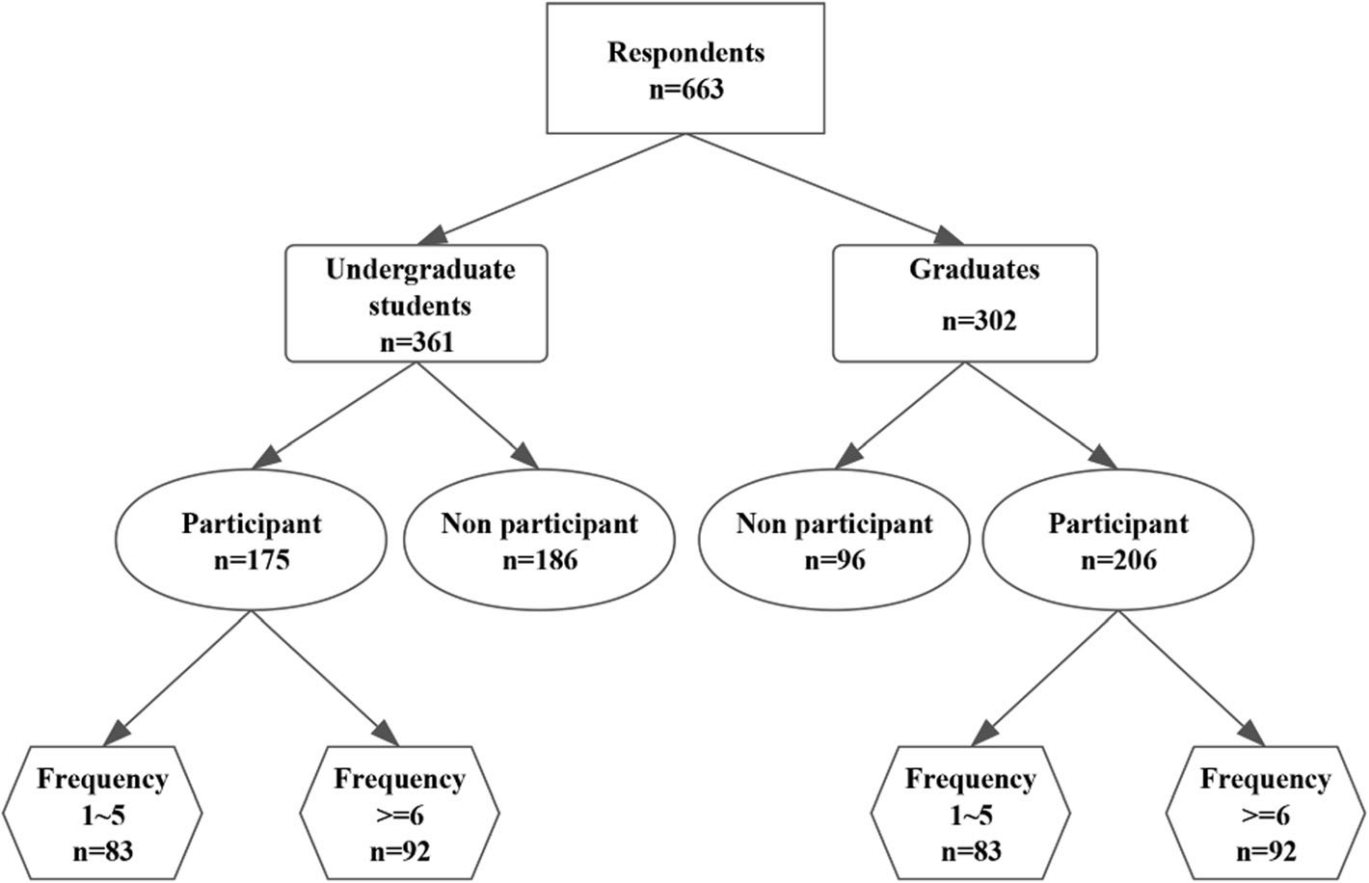
Illustration of the data collection process

This study enrolled graduate and undergraduate medical students who participated in CHS from January 2008 to July 2017, and they were invited to complete the questionnaire and receive brief interviews. A total of 876 individuals were invited to participate. Of these, 663 completed the questionnaire and attended the interviews, for a response rate of 75.6%. Out of the 663 respondents, 361 (54.4%) were undergraduate students and 302 (45.6%) graduates. Table 2 shows the demographic characteristics of the study population; no significant differences were observed between the groups. A total of 48.5% (175/361) of the students and 68.2% (206/302) of the graduates had participated in CHS programs (Fig. 1).

**Fig. 2:**
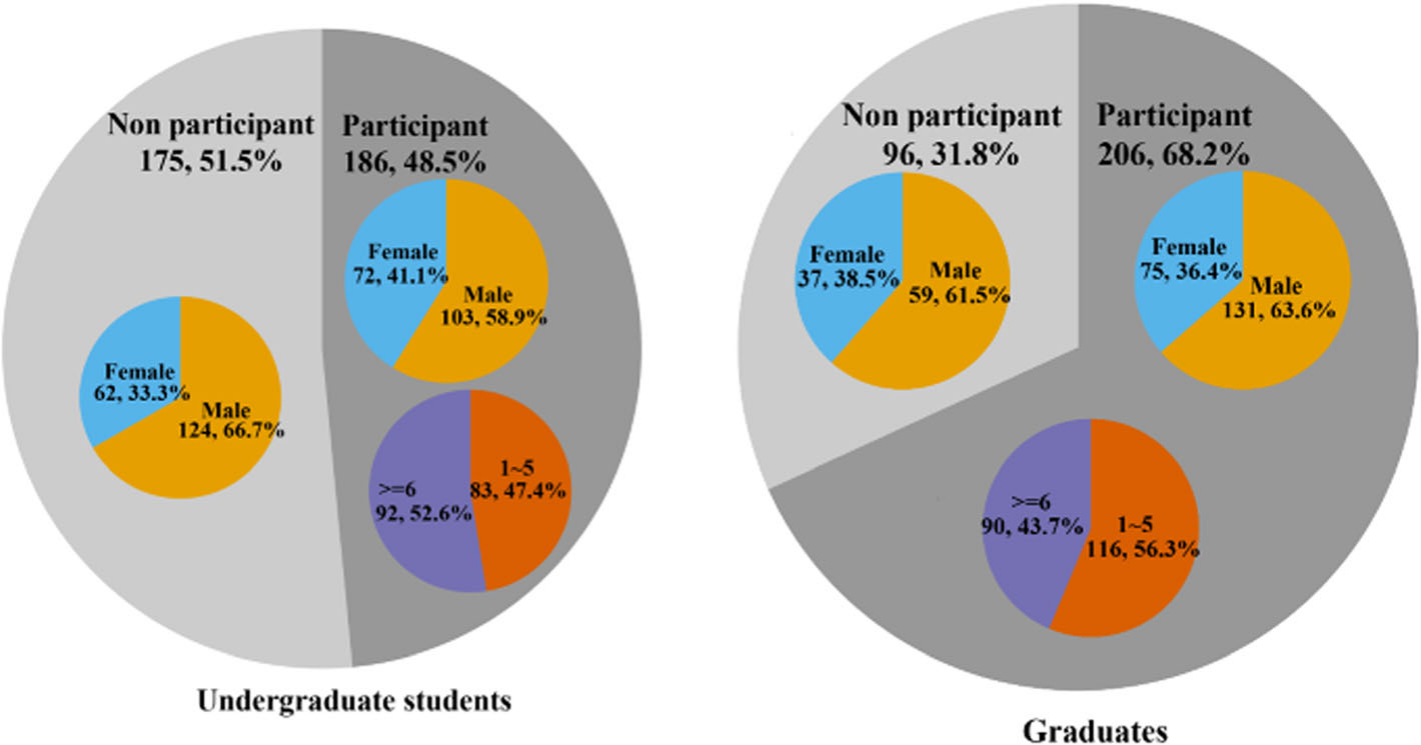
Characteristics of the study population

In the graduate and undergraduate student groups, significant differences were observed in the self-evaluation of ethical sensitivity development between those who had participated in CHS programs 1–5 times and those who had participated more than 6 times (Table 2) (Fig. 2).

**Table 2:** Characteristics of the study population

	Undergraduate Students (*n* = 361)			Graduates (*n* = 302)		
	participant	non participant	*p*	participant	non participant	*p*
	*n* = 175	*n* = 186		*n* = 206	*n* = 96	
Gender						
Male	103 (58.9%)	124 (66.7%)	0.125	131 63.6%)	59 (61.5%)	0.721
Female	72 (41.1%)	62 (33.3%)		75 36.4%)	37 (38.5%)	
Participating frequency						
1–5	83 (47.4%)			116 56.3%)		
≥6	92 (52.6%)			90 43.7%)		

“Participants” were those who participated in the CHS programs. “Nonparticipants” were those who had not participated in the CHS programs

**Fig. 3:**
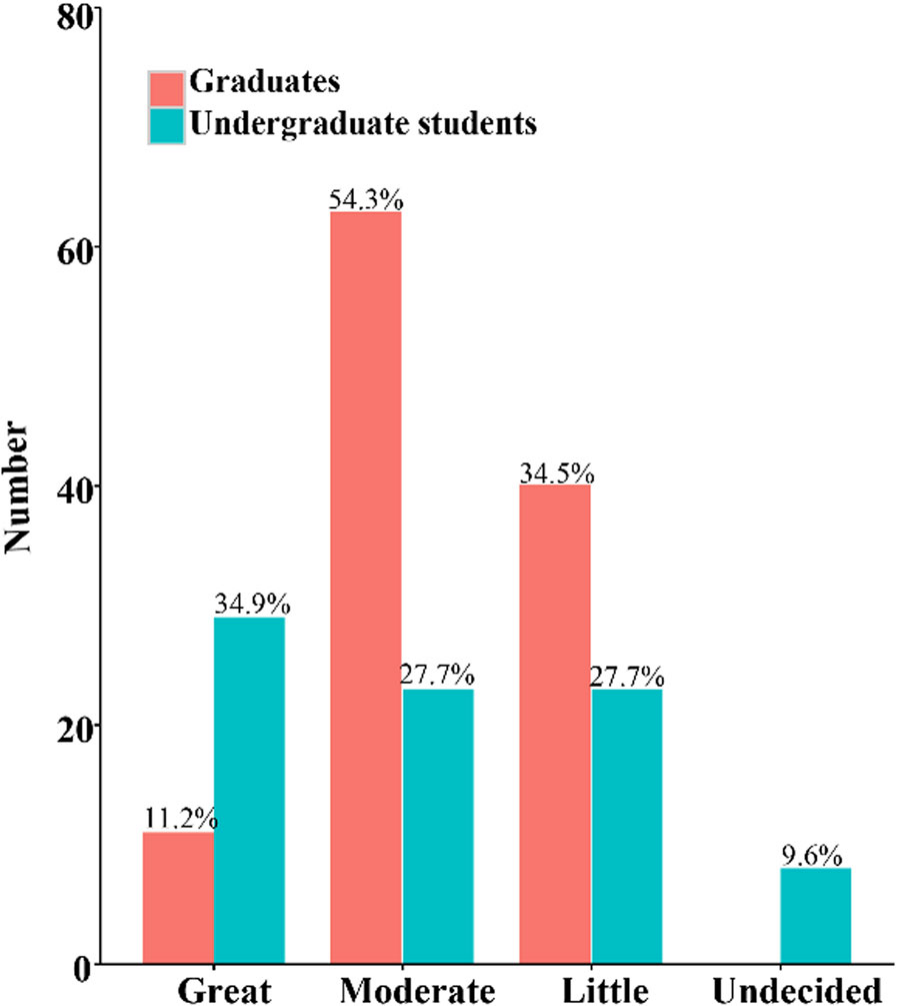
Self-evaluation of medical ethical sensitivity development by students who had participated in CHS programs 1–5 times

**Fig. 4:**
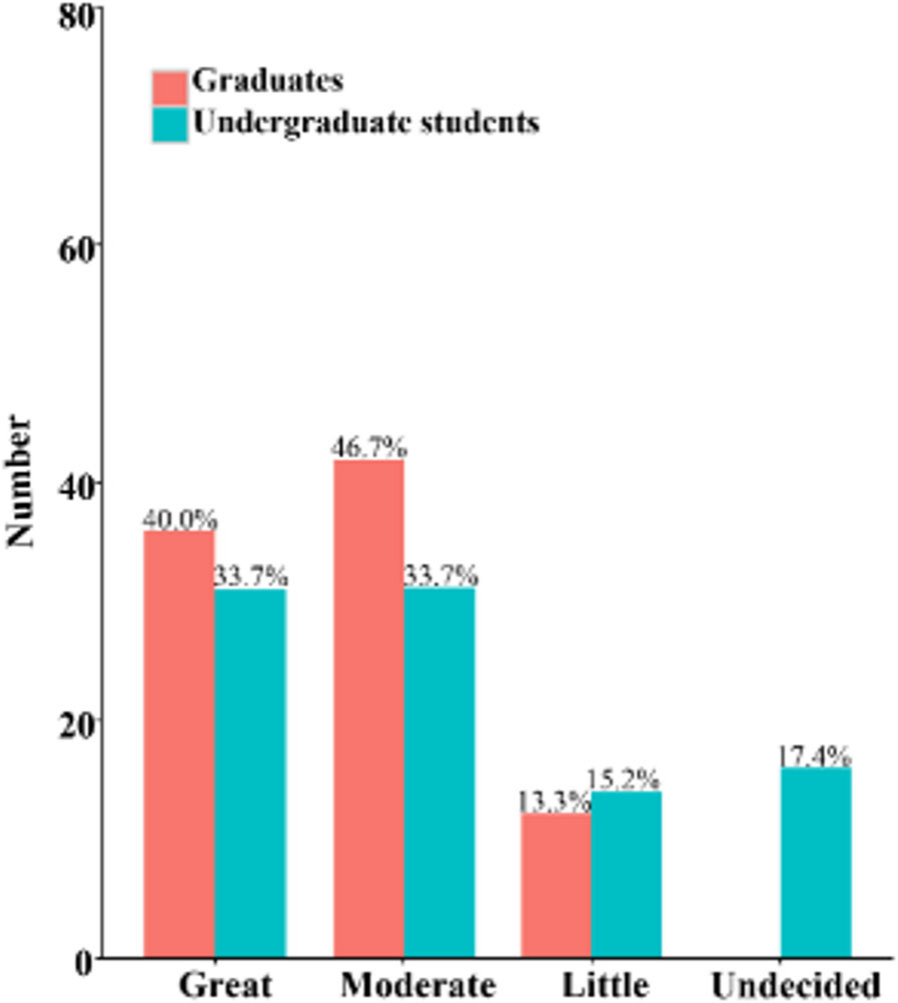
Self-evaluation of medical ethical sensitivity development by students who had participated in CHS programs more than 6 times

“Red envelope” indicates the receipt of money in addition to the normal treatment fee by physicians; this practice is unethical. Self-reports regarding the willingness to receive a red envelope significantly differed (*p* = .031) among the graduates (Table 3) but not (*p* = .567) among the students (Figs. 3 and 4).

**Table 3:** Self-evaluation of medical ethical sensitivity development

Frequency 1–5		≥6	*p*-value
Graduates			
Great	11 (11.2%)	36 (40.0%)	**< 0.001**
Moderate	63 (54.3%)	42 (46.7%)	0.276
Little	40 (34.5%)	12 (13.3%)	**0.001**
Total	106	90	
Unergraduate Students			
Great	29 (34.9%)	31 (33.7%)	0.863
Moderate	23 (27.7%)	31 (33.7%)	0.392
Little	23 (27.7%)	14 (15.2%)	**0.043**
Undecided	8 (9.6%)	16 (17.4%)	
Total	83	92	

“Great/moderate/little” indicates the respondents’ opinion that CHS had a great/moderate/little effect on their ethical sensitivity development. “Undecided” indicates that the respondents could not make a choice

*Significant level: *p* ≤ 0 .05

**Fig. 5:**
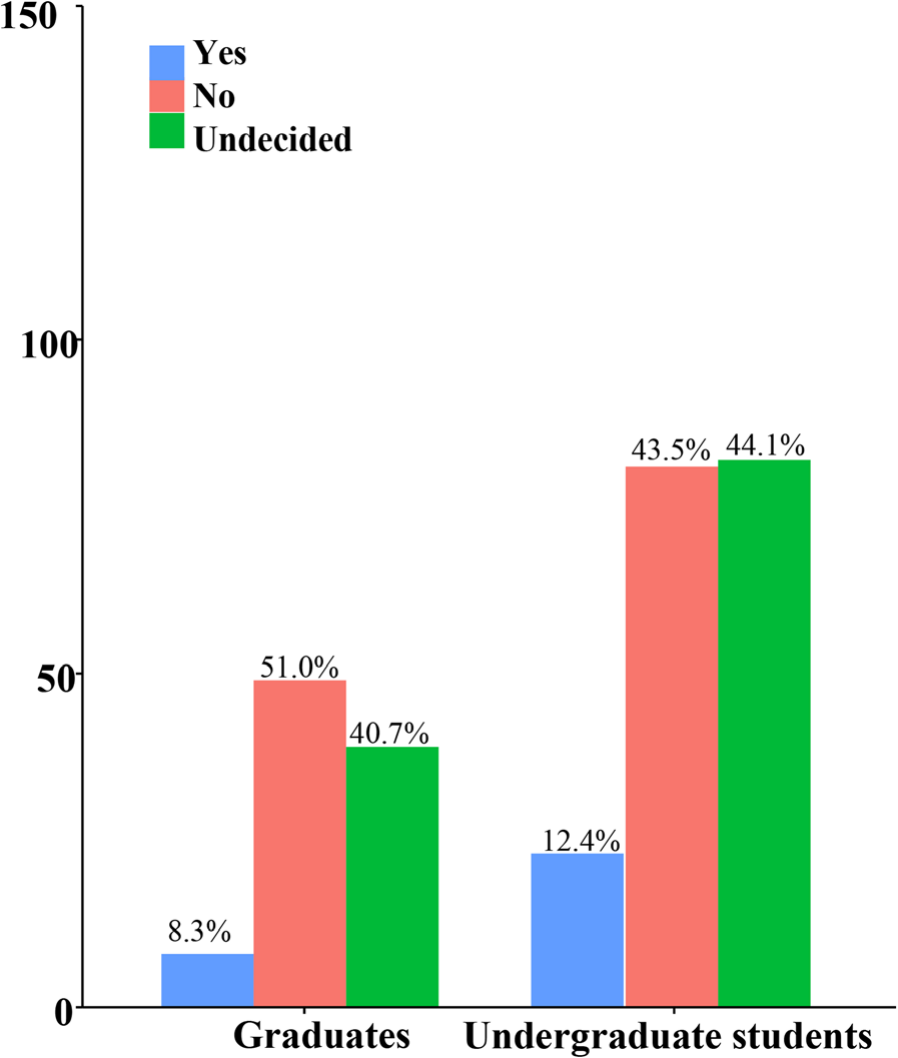
Nonparticipants’ self-report regarding the receipt of a red envelope

**Fig. 6:**
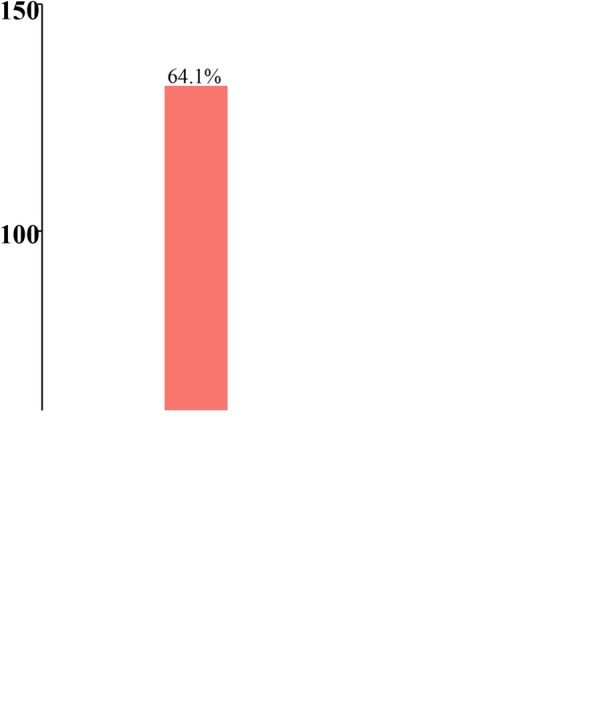
Participants’ self-report regarding the receipt of a red envelope

In addition, the researchers conducted a qualitative content analysis of the impact of CHS programs held in senior care facilities and in rural communities on the students’ cognition of medical ethics. Interviews were conducted for those (*n* = 36) who participated in the CHS programs. In the study process, qualitative interview transcripts were formatted and imported into NVIVO 10 software for data management, coding, and analysis and were then coded by the qualitative team (Figs. 5 and 6, Table 4).

**Table 4:** Node level and material information

Tree node and child node		Material source^a^	Reference point^b^
Cognition	Motivate us to study hard.	16	18
	Better understanding and compassion for patients.	19	20
	Aware of there are a lot of people suffering with chronic diseases.	17	23
	Helping others is meaningful.	12	13
	Thought preparation for being a doctor.	7	10
	To understand the status quo of rural areas.	7	7
	Aware of the importance of communication.	2	2

^a^The number of interviews with the node

^b^The source of the node in the interview material

The participants’ perspectives regarding cognitive development through CHS programs are summarized in Table 5. The views of the participants were generally very positive, as demonstrated in the following narratives:

- *Learned to transpose thinking and better understand and sympathize with the patients.*
- *Enhanced our sense of responsibility and motivated us to study hard.*
- *Enhanced our awareness of other people who need our help in society, especially those who are suffering from chronic diseases.*
- *Motivated me to call on more people for participating in volunteer service to help more people.*

**Table 5:** Self-report regarding the receipt of a red envelope

	No participant	Participant	*p*
Will you accept patients ‘red envelope’?			
Undergraduate Students			
Yes	23 (12.4%)	17 (9.7%)	0.423
No	81 (43.5%)	71 (40.6%)	0.567
Undecided	82 (44.1%)	87 (49.7%)	0.284
Graduates			
Yes	8 (8.3%)	19 (9.2%)	0.801
No	49 (51%)	132 (64.1%)	**0.031**
Undecided	39 (40.7%)	55 (26.7%)	0.015

“Participant” indicates those who had participated in the CHS programs; “nonparticipant” indicates those who had not participated in the CHS programs

*Significant level: *p* ≤ 0 .05

Accordingly, the following conclusions can be drawn: CHS activities can effectively increase students’ participation in social services. The participants expressed willingness to care for disadvantaged groups, thus promoting continued service. The single CHS programs are adapted to undergraduate students. In addition to personal life inspiration, the participants also acknowledged the benefits of the joy of service, promotion of self-value, and attention to society. These activities effectively improved the students’ involvement and commitment to serve the community, and through such a service process, the students also developed an enhanced value of the inner self, a sense of responsibility, and an understanding of life care.

## Discussion

CHS has become an important component of medical education worldwide and can be effectively integrated into the curriculum [48]. Unlike the CHS programs and research at medical colleges in other countries, those in mainland China are grossly inadequate. SUMC is the first medical college in China to introduce CHS programs as part of the medical education. It aims at improvement through exploration and constant learning from experience. CHS can be performed in different ways to reflect the multitude of community needs and adapt to the needs of students at different learning stages. All our participants spent more than 6 h per activity, which provided sufficient time for the students to understand the living plight and real needs of the poor people, as opposed to a one-to-one meeting in the clinic.

The main participants were third- and fourth-year medical students, who were in the transition stage between basic medical science and clinical training [49]. The comments of the participants were very positive and consistent, especially regarding the development in their sensitivity toward ethical cognition. Most of the participants believed that CHS had a great or moderate impact on their ethical cognition development. This indicates that CHS programs are acceptable to students in mainland China.

In China, the exchange of red envelopes has been a widespread phenomenon in medical circles [50]. This is a method of rewarding physicians for special care and of expressing appreciation. The red envelope was originally developed as a manner of expressing courtesy and respect in East Asian countries, but physicians began to abuse the practice, demanding a red envelope from patients or their families. The red envelope even became a major source of income for physicians, markedly hampering the doctor–patient relationship. Demanding a red envelope is an unethical practice and is banned in China, but it still occurs widely in private. A study revealed that 54.4% of patients [51, 52] had given a red envelope to doctors to be favored for hospitalization or surgery. Therefore, we chose the red envelope as an index for the self-report of ethical behavior. Although the ethical behavior self-report regarding the red envelope showed no significant differences (*p* = .567) among the students, significant differences were observed (*p* = .031) among the graduates. The development of ethical sensitivity and moral maturity is a gradual process and requires frequent training and practice. The CHS activities of the medical students are especially effective in identifying community patients’ unmet needs [53].

Medical ethics education is an important part of medical education, and it helps students to establish the proper values, outlook on life, and world view, which are related to medical ethical cognition and behavior [54]. Leading and promoting medical students to develop appropriate medical ethics constitute a crucial purpose of medical education and a key marker for evaluating the effectiveness of medical education. CHS exerts a subtle influence on medical students’ ethical cognition, and many participants believed that CHS had a great impact on their ethical cognition development.

The major contributions of this study are as follows.

First, we used a self-report method to verify the effectiveness of CHS for intervening in the potential regression of morality in junior medical students influenced by Chinese culture.

Second, our study verified the self-regulation theory and the effectiveness of community medical service in moral compensation. Our results suggested that through CHS activities, the students’ moral awareness was raised, which prevented them from regressing on the morality scale. In this article, this effect is termed the “prevention focus” of CHS.

Third, through the evaluation of community medical service activities, we preliminarily explain the paradox between medical students’ morality education and the regression of morality among them. According to compensation theory, unethical behavior damages the moral self-image of physicians. To restore this image, the physicians then perform some positive action as compensation. However, in reality, the morality regression phenomenon is still observed, implying that in Chinese culture, the ethical regression in a small percentage of physicians will increase in severity.

Our findings revealed that to avoid a long-term regression in medical students’ morality, strengthening their understanding of immoral behavior is essential. People with morality problems may rationalize their behavior; specifically, they do not consider their behavior immoral. In our study, the community medical service activities had a moral compensation effect. The students could first identify which behavior is moral and then could continue compensating for it.

Finally, this study supports the view that the community medical service activities had a prevention focus effect, which can deter unethical behaviors among medical students. Service activities can also activate the students’ cognition toward social medical activities and avoid a morality decline in their long-term career, which prevents immoral cognition from developing into real unethical behavior.

Several limitations of this study should be noted. The impact on cognition is a long-term process and should be monitored and evaluated constantly. We evaluated only the differences between participants and nonparticipants in medical ethics cognition and behavior self-reports through a single survey and ignored the relationship between students’ volunteer experiences and their medical school academic performance, clinical skills, and residency performance. All participation was voluntary, and those who participated were more likely to report a positive attitude toward extracurricular activities. Dynamic research requires constant follow-up. In addition, other factors potentially influencing the community students’ cognition and the community patients’ responses and satisfaction might exist [54]. Ultimately, CHS programs are at an immature stage and have not sufficiently been integrated into the medical education curriculum.

## Conclusions

In summary, CHS in mainland China may play a crucial role in medical education. The inclusion of CHS in medical education is welcomed by medical students, and our findings are a reference for promoting CHS in other medical schools in mainland China. We hope that our success in integrating this concept into medical education serves as a catalyst for the wider application of this concept by other medical universities in mainland China.

### Practice points

- The inclusion of CHS in medical education is welcomed by Chinese medical students.
- CHS has a prevention focus, which can deter unethical behaviors among medical students.
- CHS has a clear effect on ethical cognition, which should be promoted.

## Data Availability

The datasets used and analyzed during the current study are available from the corresponding author on reasonable request.

## Abbreviations

CHS: Community health service
SCT: Social cognitive theory
SPSS: The Statistical Package for Social Sciences (IBM SPSS Statistics 19.0)
SUMC: Shantou University Medical College
